# Setup of a cryobank for ovarian tissue in a university-based setting

**DOI:** 10.3389/fendo.2023.1193178

**Published:** 2023-05-25

**Authors:** Dunja M. Baston-Büst, Iwona Scheliga, Alexandra P. Bielfeld

**Affiliations:** Department of OB/GYN and Reproductive Endocrinology and Infertility (REI), UniKiD/UniCareD, Medical Hospital University of Düsseldorf, Düsseldorf, Germany

**Keywords:** cryopreservation, freezing, gonadotoxic, children, female, infertility

## Abstract

Establishing and maintaining a newly set-up cryobank for ovarian tissue in a university setting requires at least 1 year’s notice to start financial, spatial, lab equipment, and employee acquisition planning. Right before and after the start of the cryobank, the newly founded team should introduce itself to the hospitals and local and national health systems *via* mail, print flyers, and symposia in order to share the possibilities and the knowledge. Potential referrers should be provided with standard operating procedures and advice on getting used to the new system. Especially in the first year after the establishment, all procedures should be internally audited in order to avoid possible difficulties.

## Introduction

Establishing a cryobank for ovarian tissue (OT) that is technically state-of-the-art in terms of both personnel and equipment is the basic prerequisite for establishing a new medical unit at a German university hospital. This certainly requires good planning and a certain amount of lead time in order to fulfill the aforementioned prerequisites. Once these conditions have been met, however, the real work begins, because now the newly installed cryobank team must ensure that a wide variety of target groups are informed that there is now a cryobank at this location and what specific services are available for patients and medical staff.

## Planning of the setup of a centralized cryobank and financial aspects

**Figure 1:**
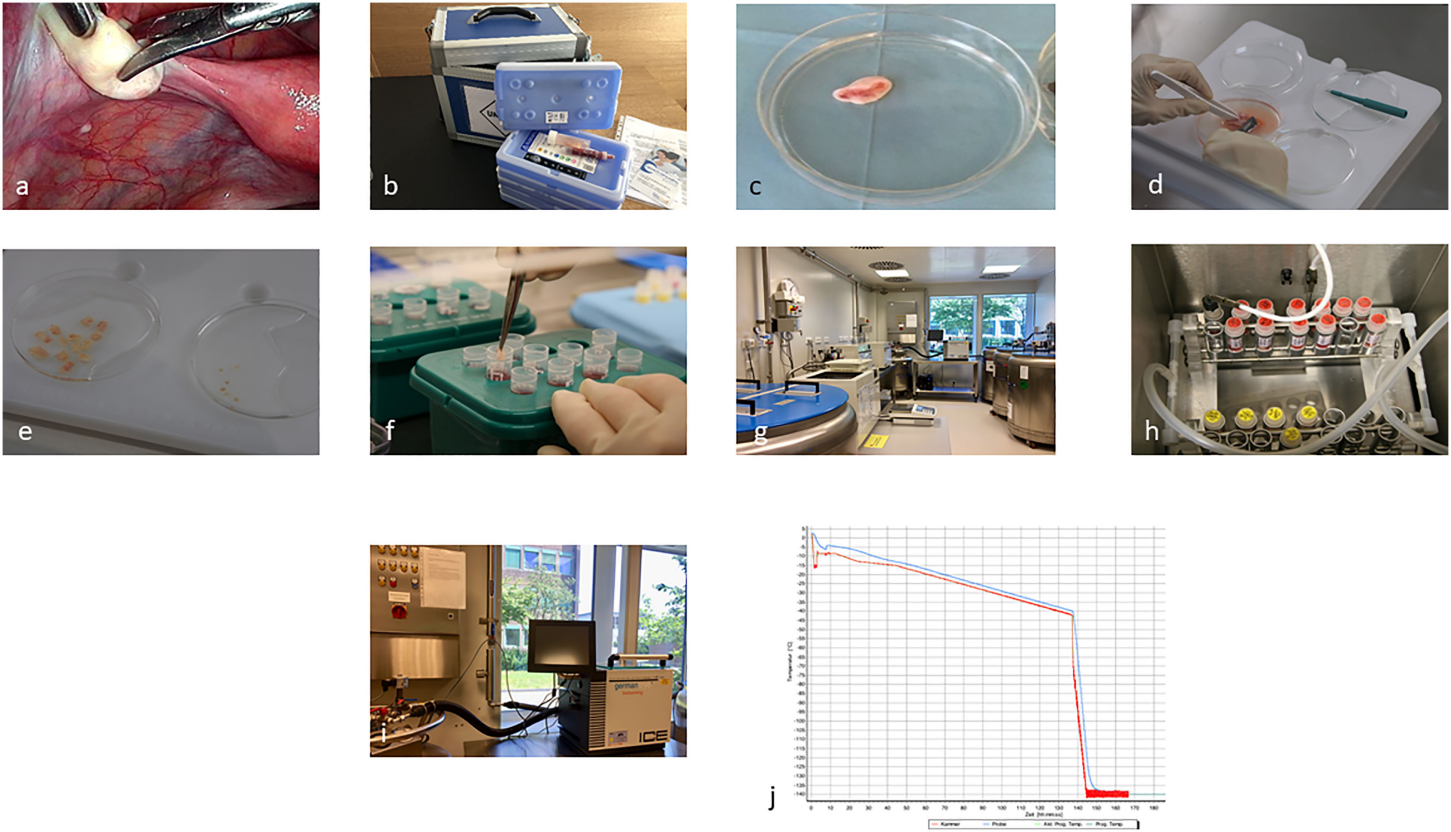
Representing the process from laparoscopic biopsy **(A)**, (overnight) transport **(B)**, and preparation and freezing of the cortical biopsies **(C–J)**.

Because of the few existing cryobanks for fertility preservation (FP) in Germany at the moment and the unproblematic dispatch of the cryomaterial, it is indispensable to engage with and become known to groups far beyond the normal catchment area. Prior to the start of a cryobank, there were many meetings with the head of the department of OB/GYN, the dean of the medical faculty, and the legal and the financial departments, which dealt with spatial possibilities, financial aspects, the design of the contracts, the scientific foci of the university hospital, and obviously the staff needed. Either new or existing staff with experience in the *in vitro* fertilization (IVF) lab or newly employed experts in the field were qualified for this position. In our case, meetings with the financial department were more fruitful as an IVF unit has been established before. Furthermore, next to the lab and storage space for the cryobank, an area for consultations should be offered within the center in the IVF unit, representing specialists in reproductive medicine (**Supplementary Figure 1**). The minimal equipment of a cryobank should be a laminar flow for the different steps during freezing and thawing, a fridge and a refrigerator for the transport and cryopreservation medium, a printer for the cryobonds, a slow freezing unit with access to liquid nitrogen, and a storage tank. As part of the public health system, the financial benefit could be more nonprofit than established as a private cryobank. A private cryobank for ovarian tissue cryopreservation (OTC) in Germany would need about 350,000€/year for its establishment and maintenance, which is more expensive than that estimated by Kyono et al. (1). In our unit, the cryobank started on the same campus in the GCP unit, focusing on blood stem cells and cord blood. In order to offer national support, numerous transport boxes and cooling elements have been bought, and a contract with a parcel service has been signed (**Figures 1A, B**). Regarding the number of staff needed on site, a minimum of two specialists should be educated to freeze and thaw OT, supported by a patient manager who responds to the patient’s questions and concerns and organizes the logistics of the boxes and the data management. About 400 patients a year perform OTC, according to the Fertiprotekt data collection and data from Japan (1, 2). The patient’s burden to pay for FP, including OTC, will be covered by the medical insurance in Germany soon. Since 2021, the costs of freezing mature oocytes for FP are covered by the insurance companies as long as the patient is over 18 years old and, in the case of breast cancer, hormone receptor negative.

## Information on referrers and potential patients

A distinction must be made here as to who the information is intended for. On the one hand, there are affected patients of reproductive age or even children with a malignant disease who will be treated with chemotherapy and/or radiation and who may be at risk of moderate to complete loss of fertility, depending on the treatment regimen (3, 4). In addition, there are patients with benign diseases, e.g., thalassämia, who are also going to receive a gonadotoxic treatment (5). Patients with genetic predisposition or severe endometriosis disease may also be considered for fertility preservation (5). All patients, especially those with an oncological disease, are already focused on treatment and survival. Due to the absence or limited possibilities for biological parenthood after successful therapy and remission for those receiving high-risk therapy, they should nevertheless be given the opportunity to consider the topic of FP. Here, the attending physician or specialist is certainly the one to provide the patient with information about FP or the address and/or contact of a specialized counseling unit. In Germany, the documentation of the patient’s education concerning FP is part of the certification prerequisites for cancer centers.

Moreover, gaining information through an Internet search is also becoming increasingly important nowadays. Here, patients inform themselves independently, so it is important to initiate the media presence at the same time during the establishment of a cryobank. An informative and easy-to-navigate website with information about the existing options, information on contact (in addition to phone contact, electronic contact is increasingly favored because this is independent of the opening hours of the center and is therefore considered a very low-threshold offer), and, in the case of the OTC and or gametes, also information on the costs and associated legally regulated requirements for cost coverage by statutory health insurance, plays an immense role. Closely related to web performance is good accessibility. This requires specially trained personnel on the phone and, if possible, an individual phone and/or mobile number that is used only for matters of fertility-preserving needs to guarantee short-term help.

Also, foundations (Deutsche Stiftung für junge Erwachsene mit Krebs, Stiftung Lebensblicke), associations, and self-help groups (Jung und Krebs e.V., Krebs-Selbsthilfe, Deutsche Krebshilfe, Frauenselbsthilfe Krebs-Bundesverband e.V., BRCA-Netzwerk e.V.-Hilfe bei familiären Krebserkrankungen, Rheuma-Liga, Endometriose Vereinigung) are playing an important role in which emotional support is offered as well as knowledge about a disease, treatment strategies, and often also information around support, applications, etc. Therefore, it makes sense for the cryobank team to introduce itself to the local foundations/self-help groups, as well as to national ones, so that they are precisely informed about the technical possibilities and options. Members of the foundations or self-help groups can then pass this knowledge safely, quickly, and objectively to those affected. It also makes sense to keep on giving presentations at patient information days (e.g., breast cancer day), at the citizens’ university, or at meetings of self-help groups and associations, as information can also be lost there due to personnel changes.

A very current approach, which is also desirable in the area of FP, is represented by the project of the so-called professional patients or patient coaches, which has been initiated at our university hospital with a special focus on oncological patients. Here, formerly ill patients act as on-site advisors for those currently seeking help and navigate them not only physically through the often jungle-like wounded localities in the clinics but also in terms of content. The information and detailed education of these patient coaches are also good multipliers for the flow of information to the patient.

In addition to patient-oriented information and the right information tools and flow for them, the training of medical professional groups is important (**Supplementary Figure 1**). Here, it must not be forgotten that not only medical personnel who care for patients with malignant diseases but also those who care for patients with benign diseases such as immune diseases and endometriosis, as well as those with genetic predispositions such as Turner syndrome, receive differentiated and, if possible, regular information through lectures, mails, and events on the topic from colleagues on site or in the area of the cryobank. Especially when a cryobank is newly established at a location, it often takes longer for this information to get around, so it is good to organize information events even before and during the cryobank’s opening to report on this increase in possibilities. From our own experience, we can state that the sole information available through clinic news, letters, and emails is not sufficient to raise an awareness of the new situation, but the personal approach has a much higher value. Especially because the field of FP is very fast developing and therefore changing, personal and regular information of all involved specialties provides a solid counseling offer according to the current S2k guideline (6). In order to meet the needs of the healthcare providers and to gain an update on the routine of cryobanks, several questionnaires have been created in the last decade emphasizing the need for specialists and best care for all patients independent from financial burdens or social impacts (7–10).

## The daily routine of a cryobank, including thawing and transplantation service

### Surgery and freezing of OTC

Immediately prior to the start of therapy, OT can be obtained by laparoscopy within 1–2 days after counseling or even the diagnosis of a malignant or benign disease (**Figures 1A-C**). All follicles are located in the OT cortex, which is dissected into at least 10 equal-sized pieces of tissue and then cooled in a controlled manner using a computer-controlled program until it is completely frozen to −140°C (11, 12) (**Figures 1C-J**). The frozen samples are then transferred directly to the cryostorage container, where they can be stored indefinitely in the nitrogen vapor phase. In our cryobank, the initial storage period according to the contract with the patient, is 1 year, which is then extended annually. In Germany, there are only three centralized cryobank and three to five smaller centers offering processing, cryopreservation, and permanent storage of OT. Only the centralized cryobanks offer overnight shipping of OT nationwide (13). Only the centralized facilities have the special equipment and trained personnel to ensure a standardized good quality, which is needed in terms of transplantation after the patient’s recovery from the disease and the wish to conceive (14, 15).

The cryobank and the clinic where the laparoscopy takes place have signed a cooperation contract and a delimitation of responsibility agreement containing the responsibilities for processing, cryopreservation and storage, and presurgery, surgery, and postsurgery measures. The goal is always to provide the patient with the best possible care and service. A close exchange between the cryobank and the patient’s clinic is very important, including the registration of a patient during or after counseling and the coordination of the shipment of the boxes for transport (13, 16, 17) (**Figure 1B**). Essential for the information of the staff on-site as well as in external clinics is a standardized procedure, which is defined in a standard operating procedure (SOP) (**Supplementary Figure 1**). This then applies to all employees of the collection clinic, the cryobank, as well as the gynecological outpatient clinic/ward, patient management as well as the OR, and consequently all persons involved in the OT collection, preparation, and storage. The persons responsible for the various tasks should be named in the SOPs with their contact data and should receive regular training. Annual random internal audits are required. A physician is responsible for the initial interview with the potential patient, including the patient’s history, which is also possible *via* e-consult in terms of a pandemic, for example (10). The patient must be informed and advised about the expected gonadotoxicity of the therapy and the different options for FP (**Supplementary Figure 1**). Basic gynecological sonography, blood sampling for AMH, and serology for possible infectious diseases must be performed before the laparoscopy is scheduled. The cryobank provides the collection clinic with all relevant documents, such as patient information on cryopreservation, a contract, and the cost agreement (**Supplementary Figure 1**). The referring external clinics receive a checklist from the cryobank (**Supplementary Figure 1**). During the counseling about the partial/complete OT removal by laparoscopy, a possible transposition of the ovaries and potential contraindications, as well as a combination with port access, must be clarified. Important for the processing and storage of the OT in the cryobank are negative results concerning the infection parameters that need to be present 7 days before surgery. According to the German Medicinal Product Act, the Tissue Act, and the Pharmaceuticals and Active Agent Manufacturing and Distribution Ordinance, this includes anti-HIV ½ p24 antigen, HBs-Ag, anti-HBc (in case of positivity: HBV-PCR), anti-HCV-IgG, TPHA, and in case of stay in the ZIKA area in the last 6 months, the ZikaV-IgG and ZikaV-IgM. On the day of the surgery, it is the responsibility of the clinic to check the records for completeness. The cryobank checks the consent form for cryopreservation and cost coverage on the day of arrival. On the day of the surgery, a special transport box is provided by the cryobank with appropriate transport medium and nonfrozen refrigeration units (**Figure 1B**). The transport temperature should be between 4° and 8°C, and the transport time should not be more than 24 h (13). The surgeon must be trained to avoid an ovary with an active corpus luteum. If no corpus luteum is present, partial or complete removal of the ovary is preferred on the left side in Germany, preferably in one piece (**Figure 1C**) (15, 18). Any additional slicing of the ovary should be avoided, as should coagulation. Follicles are extremely temperature-sensitive to higher temperatures than 37°C. Therefore, coagulation will destroy several millimeters of the OT cortex, resulting in the loss of the embedded follicles. The removed ovary is placed directly into the cooled transport medium. Custodiol ^®^ (Dr.Franz Köhler Chemie GmbH, Bensheim, Germany) is used as our medium of choice. This organ perfusion medium is pH-stable and prevents ischemic processes in the tissue, even after 24 h at 4°C. Furthermore, a biopsy of the ovary must be sent to the pathology department of the clinic. The cryobank collects information about the surgery and, later, the results from the pathologist. Once the ovary is removed and embedded in Custodiol^®^, the temperature should remain between 4°C and 8°C, including the processing of the tissue (**Figures 1C-F**). The preparation of the ovary is performed under sterile conditions under laminar flow. It is important to avoid contamination of the OT during storage and later transplantation. The ovary section is processed in fresh Custodiol^®^ with sterile forceps, scalpels, and biopsy punch under laminar flow in a 100-mm dish on a cooling plate (**Figure 1D**). The stroma of the ovary is removed by scratching with a scalpel (**Figure 1D**). The goal of the dissection is to expose the cortex as isolated as possible, without portions of blood vessels, corpus luteum, or other tissues (11). Rectangular pieces 5–10 × 3–6 mm in length are cut from the cortex (**Figure 1E**). After completion of the dissection, all pieces are equilibrated in a sterile vessel with 10 ml of cryomedium. Currently, cryomedium is not commercially available. The formulation we use for the freezing process of the OT corresponds to the composition of Roger Gosden’s medium. In order to assess the vitality of the tissue before freezing and after subsequent thawing before transplantation, 3 × 2-mm biopsies are obtained by punching from different regions of the cortex (**Figures 1E**, **Supplementary Figure 2**). The so-called vitality test is performed after digestion with collagenase and subsequent incubation with calcein (Thermo Fisher Scientific, Meerbusch, Germany). The vital follicles can be examined by fluorescence microscopy (19) (**Supplementary Figure 2**). The entire freezing process with a slow freezing method and automatically induced seeding takes about 2.5 h and is recorded (**Figures 1G-J**). Samples are first progressively cooled to −40°C and then to −140°C before being transferred to liquid nitrogen for long-term storage. In an electronic management system for the cryopreserved materials, an overview of all incoming and outgoing samples can be obtained.

### Thawing service

**Figure 2:**
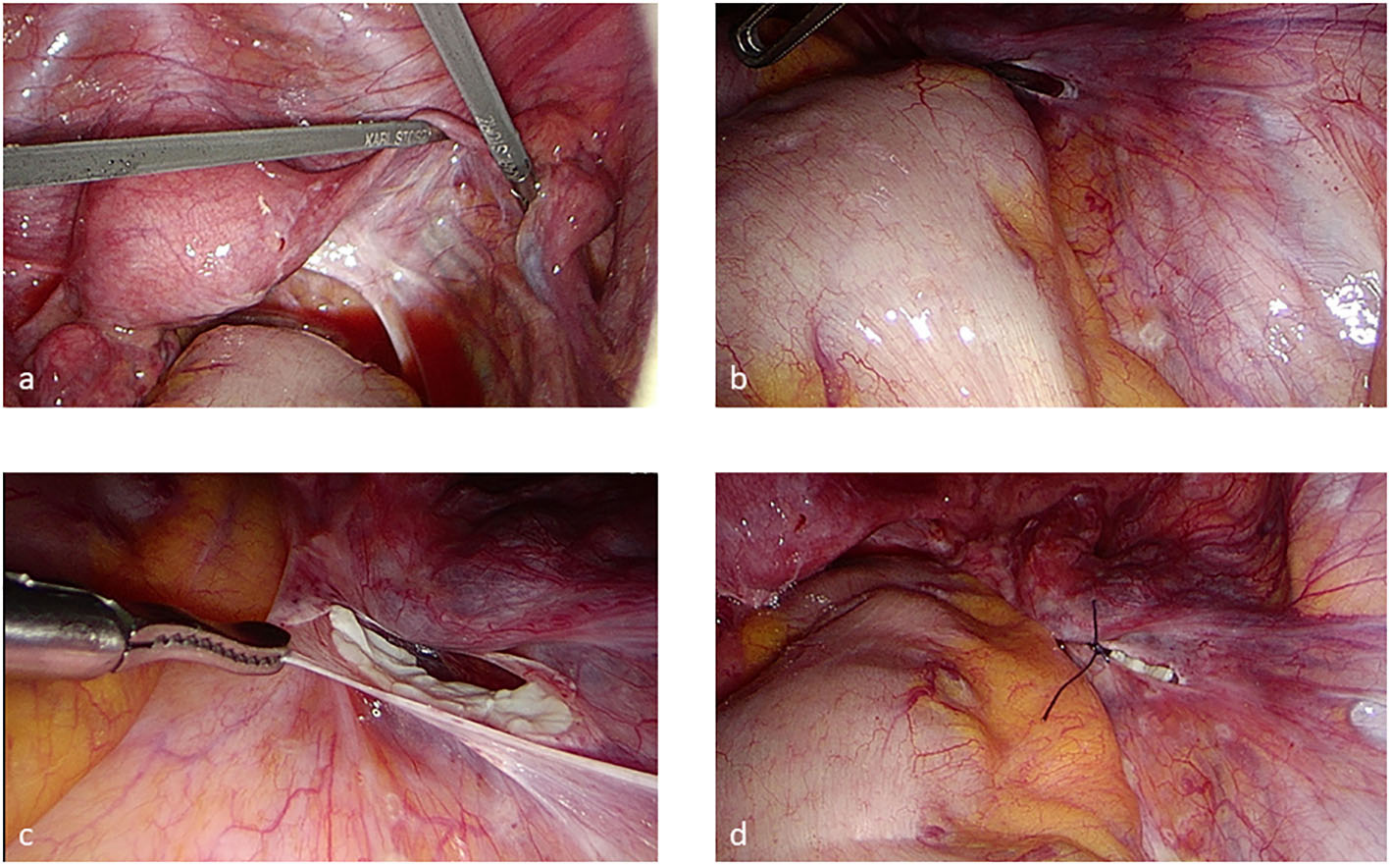
Transplantation in a peritoneal pocket by laparoscopy **(A–D)** after chromopertubation. The pieces should be transplanted on the right side if this is possible to simplify ovum pick-up if the patient needs an ART treatment.

At our location, we have also established the offer of the so-called mobile thawing service for OT, so that the respective clinic, which has removed a tissue, can transplant it later (17). Nevertheless, most transplant patients are cared for in a few specialized clinics with well-trained surgeons. The date of the OT is organized by the respective clinic, the patient, with the oncologist’s informed consent, and the cryobank. A sterile hood for the thawing process should be available within a 10-min walk from the surgery theater. The well-trained staff of the cryobank and the frozen patients’ tissues move to the respective clinic. The thawing process takes about 60 min. All media and technical devices are transported as well. Before slicing the peritoneal pocket, the fallopian tubes are checked for perturbation to enable spontaneous pregnancy. The transplantation in a peritoneal pocket and the thawing procedure are coordinated in a timely manner (**Figures 2A-D**) so that the ovarian grafts are transplanted as soon as possible after thawing. Previously, the grafts were orientated with the outer cortex toward the tube. Recently, Kristensen et al. reported that revascularization does not need this orientation, which reduces the time of the surgery as well (20).

## Discussion

For the establishment of a cryobank for OT in a university setting, some prerequisites need to be fulfilled: lead time, well-educated and dedicated staff, on-site space, and a financial plan covering the costs of the establishment and the possible outlay for the first 5 years. A major strength of the Faculty of Medicine at the UKD lies in the interest in and the priority research field of oncology and the establishment of state-of-the-art treatment options. Since 2013, the University Tumor Center (UTZ) has been certified as an Oncology Center of the German Cancer Society (Deutsche Krebsgesellschaft (DKG)), and from 2014, oncology at the university hospital was funded as a top center of the German Cancer Aid (Deutsche Krebshilfe). Furthermore, the Düsseldorf School of Oncology (DSO) supports clinical education and translational research in different disciplines, e.g., hematology and oncology, pediatrics, urology, and gynecology. Within the framework of the Centre for Integrated Oncology (CIO^ABCD^—Aachen, Bonn, Cologne, and Düsseldorf), the four sites collaborate intensively to improve patient care using combined and personalized approaches, e.g., molecular tumor boards. In order to advance treatment success and care for the future, FP for patients of reproductive age as well as children is part of the quality assessment of the DKG. We aim to cross interdisciplinary and regional boundaries through education and knowledge exchange in order to improve patient care. We would like to strengthen and extend innovative, interdisciplinary clinical and scientific training structures and platforms to facilitate a novel holistic view of FP concepts, including more than oncologic disciplines, e.g., ethics, psychosomatics, public health, and law.

A culture of staff working to their full potential and treating patients with compassion and understanding is very important during this stressful time for the patients and their relatives. Due to the recurring ethical and legal complexities of diverse aspects of FP (e.g., reproductive autonomy, trans/nonbinary person, cost coverage), there has also been close cooperation with the institutes of ethics and the law school for many years. Concerning quality assessment, it might be an option to design a survey for the patients who cryopreserved and stored their tissue in our cryobank (21). Furthermore, referrals can be actively surveyed, e.g., every 2 years, in order to optimize the service of the cryobank. Possible foci might be the availability *via* mail or phone, the costs, and the design of the flyers.

## Conclusion

The development of a corporate professional identity is an essential instrument in the healthcare environment in order to create a positive and unique impression on the patient and the referring professionals. Nowadays, the patient has a wide range of options in outpatient care available to them compared to former times when the first baby was born after freezing and thawing of OT (22). This has also changed the role of the physician in society. The physician now has to develop organizational and business abilities in addition to providing advice, care, and healing for the patient. Hence, the importance of translation and transfer from basic research to clinical application and comprehensive healthcare will continue to increase and should improve patient care worldwide.

## Data availability statement

The original contributions presented in the study are included in the article/**Supplementary Material**. Further inquiries can be directed to the corresponding author.

## Author contributions

DB-B directed the project; DB-B, IS, and AB wrote the article. All authors contributed to the article and approved the submitted version.

## Supplementary material

Supplementary Figure 1 A friendly environment and a sufficient timeslot in the schedule should be offered for counseling.

Supplementary Figure 2 Vitality testing using immunofluorescence staining with calcein **(A, B)**. **(C-E)** Images of follicles with different sizes (primary to secondary follicles). The shallow green dots represent already atretic follicles in the biopsy.
