# Supplementary figures and images for: Setup of a cryobank for ovarian tissue in a university-based setting
Source: Front Endocrinol (Lausanne). 2023 May 25;14:1193178. doi: 10.3389/fendo.2023.1193178 (PMC10248428; doi:10.3389/fendo.2023.1193178)

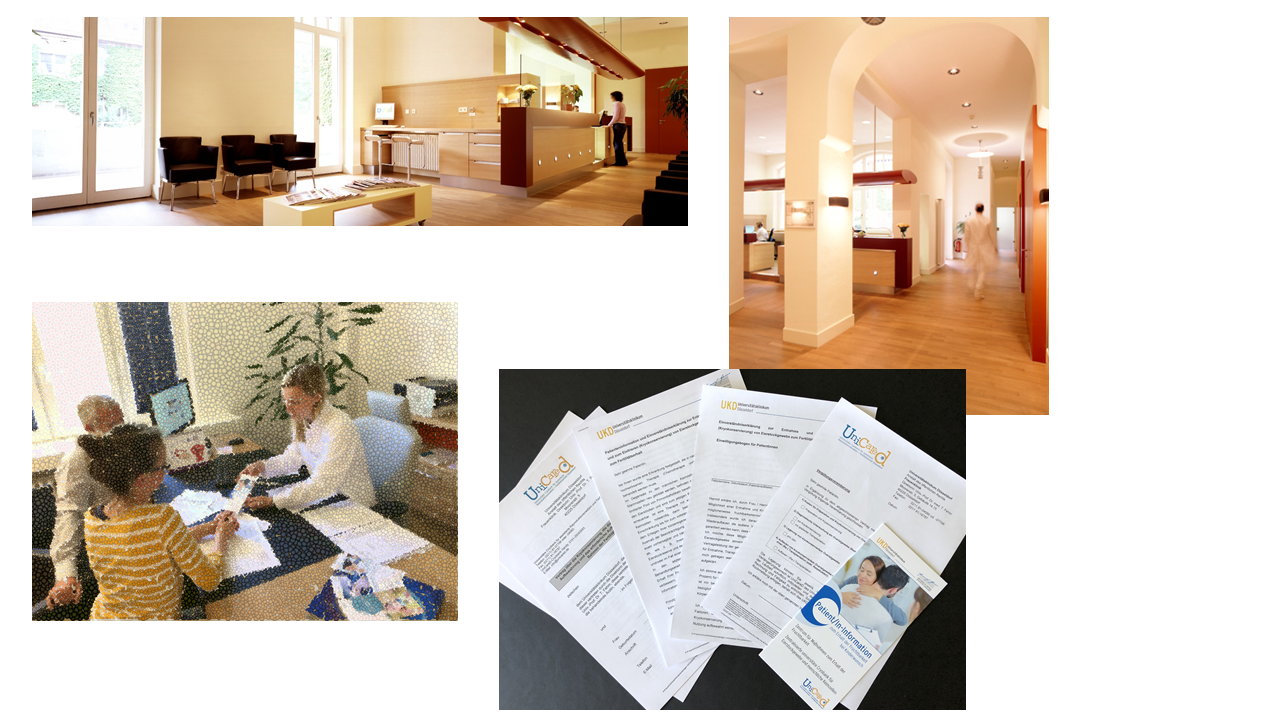

Supplement: Supplementary Figure 1 — A friendly environment and a sufficient timeslot in the schedule should be offered for counseling. [file Image_1.tif]

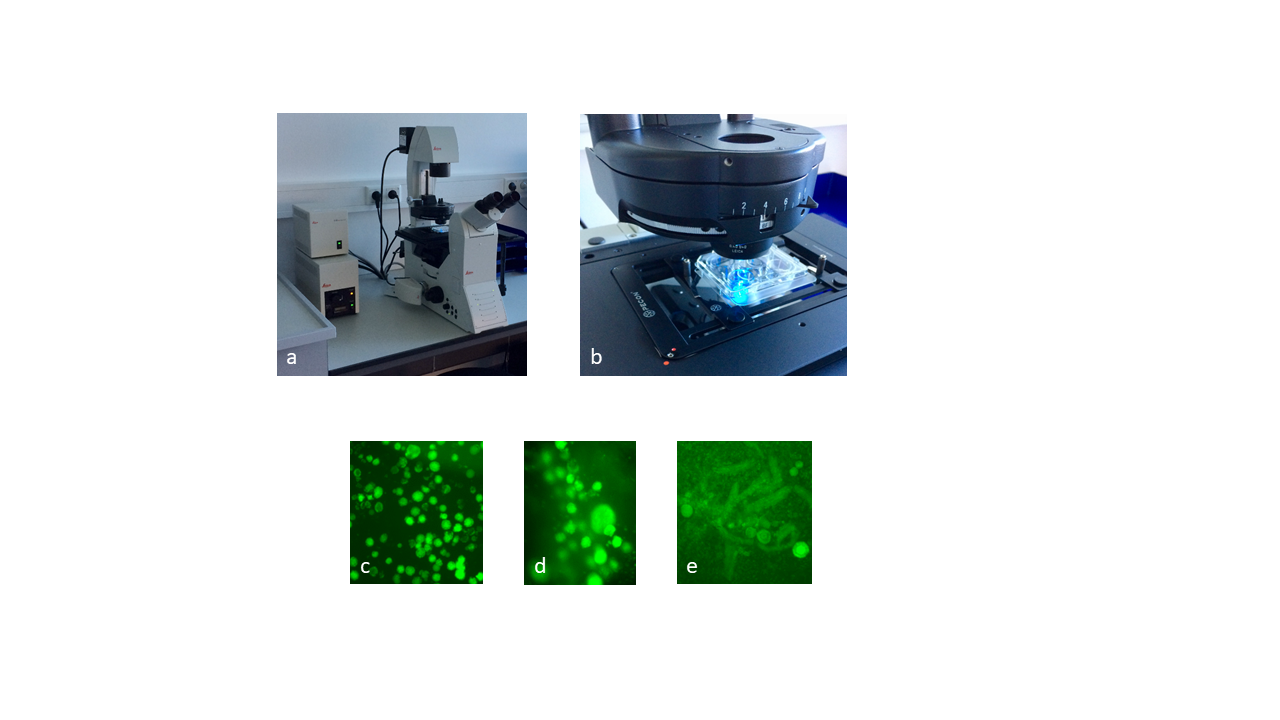

Supplement: Supplementary Figure 2 — Vitality testing using immunofluorescence staining with calcein (A, B). (C-E) Images of follicles with different sizes (primary to secondary follicles). The shallow green dots represent already atretic follicles in the biopsy. [file Image_2.tif]
